# Relationship of Sleep Quality and Health-Related Quality of Life in Adolescents According to Self- and Proxy Ratings: A Questionnaire Survey

**DOI:** 10.3389/fpsyt.2012.00076

**Published:** 2012-09-03

**Authors:** Karolin Roeser, Ruth Eichholz, Barbara Schwerdtle, Angelika A. Schlarb, Andrea Kübler

**Affiliations:** ^1^Department of Psychology I, University of WürzburgWürzburg, Germany; ^2^Faculty of Science, Department of Psychology, University of TübingenTübingen, Germany; ^3^Department of Clinical Psychology and Psychotherapy, University of Koblenz-LandauLandau, Germany; ^4^Institute of Medical Psychology and Behavioral Neurobiology, University of TübingenTübingen, Germany

**Keywords:** quality of life, sleep, adolescence, parent-child agreement, sleep disorders

## Abstract

**Introduction:** Sleep disturbances are common in adolescents and adversely affect performance, social contact, and susceptibility to stress. We investigated the hypothesis of a relationship between sleep and health-related quality of life (HRQoL), and applied self- and proxy ratings. **Materials and Methods:** The sample comprised 92 adolescents aged 11–17 years. All participants and their parents completed a HRQoL measure and the Sleep Disturbance Scale for Children (*SDSC*). Children with *SDSC T*-scores above the normal range (above 60) were classified as poor sleepers. **Results:** According to self- and proxy ratings, good sleepers reported significantly higher HRQoL than poor sleepers. Sleep disturbances were significantly higher and HRQoL significantly lower in self- as compared to parental ratings. Parent-child agreement was higher for subscales measuring observable aspects. Girls experienced significantly stronger sleep disturbances and lower self-rated HRQoL than boys. **Discussion:** Our findings support the positive relationship of sleep and HRQoL. Furthermore, parents significantly underestimate sleep disturbances and overestimate HRQoL in their children.

## Introduction

Compared to sleep patterns in childhood, sleep in adolescence is characterized by less slow-wave-sleep and lower REM density (Dahl and Lewin, 2002). Furthermore, the circadian preference at puberty changes to later bed and rising times, i.e., eveningness (Crowley et al., 2007), which is incompatible with the early beginning of school on weekdays. Social changes, for example less parental control and more peer-group activities, also cause later bedtimes in adolescents (Dahl and Lewin, 2002). Thus, biological and psychosocial alterations at puberty lead to decreased sleep depth and duration and can cause increased daytime sleepiness.

Many empirical findings demonstrate that adolescents are often sleep deprived, specifically on weekdays. In different adolescent samples, 25–50% state that they would require more sleep and frequently experience daytime sleepiness (Strauch and Meier, 1988; Morrison et al., 1992; Oginska and Pokorski, 2006; Gaina et al., 2007). This lack of sleep-related physiological restoration may result in adverse effects on school performance (Fallone et al., 2005) and on the susceptibility to stress (Roberts et al., 2002). Mnemonic and attention deficits are likely to occur (Steenari et al., 2003; Millman, 2005), as well as behavioral and emotional problems (Yen et al., 2010). Due to these many negative consequences of poor sleep, adolescents’ quality of sleep might be associated with their quality of life (QoL), more precisely health-related quality of life (HRQoL). HRQoL can be defined as a multidimensional construct pertaining to the physical, emotional, mental, social, and behavioral components of well-being and function as perceived by the individual and/or observers (Bullinger, 1991). It is measured by evaluating contentment as to different domains, of which the overall construct is made up (Solans et al., 2008). Research has proven that HRQoL can be measured reliably and validly in children and adolescents (Harding, 2001), for example with the *KINDL*-Questionnaire (Ravens-Sieberer and Bullinger, 1998b). The mental health module BELLA within the German Health Interview and Examination Survey of Children and Adolescents (KiGGS), provides HRQoL (measured with the *KINDL*) in different age groups within a non-clinical sample (Ravens-Sieberer et al., 2008). Self-reported HRQoL decreases significantly between the age of 11 and 17, except for the “self-esteem” subscale, on which scores rise. Adolescent girls (14–17 years) report lower HRQoL than boys, except for the school-related dimension. Those trends are also visible in parental HRQoL-ratings of their children’s HRQoL.

A comprehensive literature survey in Bullinger and Ravens-Sieberer (1995) revealed that children and adolescents were subject to only 13% of QoL-studies published until that time. Of these, 78% dealt with oncology and transplant medicine. An update in Gerharz et al. (2003) identified over 30,000 publications relevant to QoL in medicine, of which only 12% were related to children and adolescents. Less than 10% of the identified empirical studies included self-rated QoL. Instead, parents or clinic staff reported on the child’s QoL. Up to date, QoL research in children and adolescents has broadened across clinical samples with different diseases and chronic conditions, e.g., diabetes, asthma, and cardiac or gastrointestinal conditions (Varni et al., 2007). These groups, as well as adolescents suffering from migraine (Powers et al., 2003), obesity (Schwimmer et al., 2003), or chronic pain (Hunfeld et al., 2001), report impaired QoL compared to healthy individuals.

While associations between sleep disturbances and QoL have been found in adults with and without chronic diseases (Iliescu et al., 2003; Yoshimura et al., 2009; Eyigor et al., 2010) and in children (Hiscock et al., 2007; Quach et al., 2009), only few data are available for adolescents. Often, studies include large age groups from 5 to 18 years and are based on parental judgments only (Hiscock et al., 2007; Quach et al., 2009). However, HRQoL is a time sensitive construct as it decreases with age (Ravens-Sieberer et al., 2008). Thus, an average across a broad age range may not provide comprehensive information for adolescents. Furthermore, the parents’ perspective is not a sufficient source of information. Studies including parents’ and children’s rating of HRQoL provide moderate correlations only (Ravens-Sieberer and Bullinger, 1998a; Jokovic et al., 2004). Better agreement is found for observable (e.g., physical) compared to non-observable (e.g., emotional) aspects (Eiser and Morse, 2001). However, contradictory results exist depending on sample characteristics and on the QoL-measure applied (Upton et al., 2008). In healthy populations, parents overestimate their child’s QoL (Bullinger et al., 2008) and the moderate agreement seems not to be modified by children’s age or gender (Eiser and Morse, 2001). Consequently, when investigating QoL in children and adolescents, both ratings have to be included.

A comparison of the psychometric properties between the self and proxy *KINDL*-version demonstrates that both enable a reliable assessment of HRQoL in children and adolescents (Erhart et al., 2009). The overall correlation between parent and child ratings is .49, but differs remarkably (0.24–0.51) between subscales (Bullinger et al., 2008), supporting the mandatory inclusion of both assessments also when using the *KINDL*. The same as for HRQoL holds true for the assessment of sleep behavior in adolescents (Schwerdtle et al., 2010). Parents tend to underestimate their child’s sleep problems, specifically sleep onset latency, night wakings, and body pains during the night (Owens et al., 2000; Paavonen et al., 2000).

The aim of the present study was to examine the association of sleep quality and HRQoL in an adolescent sample. We expected an inverse relationship between sleep problems and HRQoL with poor sleepers reporting lower HRQoL than good sleepers. We further predicted moderate correlations between self- and proxy ratings and expected parents to underestimate their children’s sleep problems and to overestimate their HRQoL. According to previous results, we hypothesized lower correlations for aspects not readily accessible by the parents. Focusing on a limited age range, we did not expect age-related effects on sleep and HRQoL.

## Materials and Methods

Material consisted of a cover letter, a letter of informed consent, questionnaires (see Questionnaires), and a form for background information (including age, height, weight, presence or absence of diseases, medication, and family background). Participants were recruited from schools, youth centers, and sports clubs in Berlin, which were situated in socio-economically different districts. A total of 111 families participated, but 19 records had to be excluded due to incomplete data. All participating families gave written informed consent prior to taking part in the study, which was conducted in accordance with standard ethical guidelines as defined by the Declaration of Helsinki (World Medical Association) and approved by the ethical review committee of the University of Würzburg.

### Questionnaires

#### *Kiddo-KINDL* (Ravens-Sieberer and Bullinger, 1998b)

The *Kiddo-KINDL* is constructed for children aged 8–16 years. An analog version for their parents is available. Both consist of 47 items that have to be answered on a 5-point ordinal scale. HRQoL is assessed on six subscales (physical well-being, emotional well-being, self-esteem, family, friends, and everyday functioning in school). Transformed scores can be derived ranging from 0 to 100 on an interval scale. The empirical evaluation of the *Kiddo-KINDL* provides good reliability (Cronbach’s α = 0.92) and acceptance among adolescents and parents. Intercorrelations between adolescents’ and parents’ ratings are moderate (*r* ≈ 0.40; Ravens-Sieberer and Bullinger, 1998b). Its good construct validity is also confirmed (Harding, 2001; Solans et al., 2008).

#### Sleep disturbance scale for children (Bruni et al., 1996)

The self- and proxy version of the sleep disturbance scale for children (*SDSC*) both comprise 26 items, which are rated on a 5-point Likert-type rating scale. They assess on six subscales the most common areas of sleep disorders in childhood and adolescence: Disorders of initiating and maintaining sleep, sleep breathing disorders, disorders of arousal/nightmares, sleep wake transition disorders, disorders of excessive somnolence, and sleep hyperhidrosis. Bruni et al. (1996) report high internal consistency in healthy individuals (α = 0.79) and in children with sleep disorders (α = 0.71). They also provide normative data (*T*-values, *M* = 50, SD = 10).

### Data preparation

Subjects were defined as “poor sleepers” when their *SDSC* total *T-*value was above 60, i.e., above one standard deviation over the mean. Participants with *SDSC* total *T-*values in the normal range (i.e., lower than 60) were classified as “good sleepers.”

### Data analysis

All analyses were conducted with SPSS Statistics 18 (IBM Deutschland GmbH, Ehningen). Parental and self-rated *SDSC-* and *KINDL*-scores were normally distributed (all Kolmogorov–Smirnov-Tests ns.). Mean comparisons between groups were calculated with univariate analyses of variance, comparisons regarding frequencies with χ^2^-tests, and comparisons between self- and proxy ratings with paired *t*-tests. All correlations were calculated according to Pearson. Correlations are considered low for coefficients ≥ 0.30, moderate for coefficients ≥ 0.50, and high for coefficients ≥ 0.70 (Cohen and Holliday, 1982). We took account of confounding effects of age and sex by controlling them statistically.

## Results

The sample comprised *N* = 92 adolescents [*n* = 50 (54.3%) girls, *n* = 42 (45.7%) boys] aged 11–17 years (*M* = 13.67, SD = 1.34). While *n* = 60 (65.2%) adolescents lived with both parents, *n* = 28 (30.4%) lived with their mothers, *n* = 1 (1.1%) lived with their father, and *n* = 3 (3.3%) made no specification. Of the fathers, *n* = 77 (83.7%) had a job, *n* = 1 (1.1%) were unemployed, and *n* = 14 (15.2%) made no specification. Of the mothers, *n* = 78 (84.8%) had a job, *n* = 1 (1.1%) were housewives, and *n* = 13 (14.1%) made no specification. Regarding education, *n* = 4 (4.3%) fathers and *n* = 2 (2.2%) mothers had not finished school, while *n* = 10 (10.9%) fathers and *n* = 3 (3.3%) mothers did not indicate their educational level.

Parents’ and children’s *KINDL-*ratings correlated highly (*r* = 0.76), ranging from 0.50 (“Friends”) to 0.77 (“Physical well-being”) on the subscales. Parents’ and children’s *SDSC*-ratings correlated moderately (*r* = 0.59), ranging from 0.35 (“Sleep breathing disorders”) to 0.64 (“Disorders of initiating/maintaining sleep”) on the subscales. Partial correlations between self- and proxy ratings with the effect of age removed produced the same results. All correlation coefficients of self- and proxy ratings are listed in Table 1.

**Table 1:** **Pearson correlation coefficients of self- and proxy ratings**.

*r* (Partial *r* controlled for age)	High^a^	Moderate^b^	Low^c^
**KINDL**			
Physical well-being	0.77 (0.77)		
Total	0.76 (0.76)		
Family	0.70 (0.70)		
Self-esteem		0.61 (0.64)	
Everyday functioning		0.59 (0.58)	
Emotional well-being		0.57 (0.57)	
Friends		0.50 (0.48)	
**SDSC**			
Disorders of initiating/maintaining sleep		0.64 (0.65)	
Total		0.59 (0.59)	
Disorders of excessive somnolence		0.57 (0.56)	
Sleep wake transition disorders			0.49 (0.49)
Sleep hyperhidrosis			0.38 (0.39)
Disorders of arousal/nightmares			0.37 (0.39)
Sleep breathing disorders			0.35 (0.35)

*^a^Coefficients ≥ 0.70*.

*^b^Coefficients ≥ 0.50*.

*^c^Coefficients ≥ 0.30*.

### Good and poor sleepers

To investigate the relation between sleep quality and HRQoL, the two measures were correlated and groups of poor and good sleepers were compared. Pearson’s correlation coefficients for *KINDL-* and *SDSC-*scores were *r* = −0.48 (explained variance: 23%) in the parents’ versions and *r* = −0.36 (explained variance: 13%) in the self-ratings. Table 2 displays group sizes of good and poor sleepers depending on whose ratings were used for classification.

**Table 2:** **Group sizes of good and poor sleepers**.

Self**-**ratings	Parents’ ratings		
	Good sleepers	Poor sleepers	Total
Good sleepers	*n* = 36	*n* = 3	*n* = 39 (42.39%)
Poor sleepers	*n* = 30	*n* = 23	*n* = 53 (57.61%)
Total	*n* = 66 (71.74%)	*n* = 26 (28.26%)	

**Figure 1:**
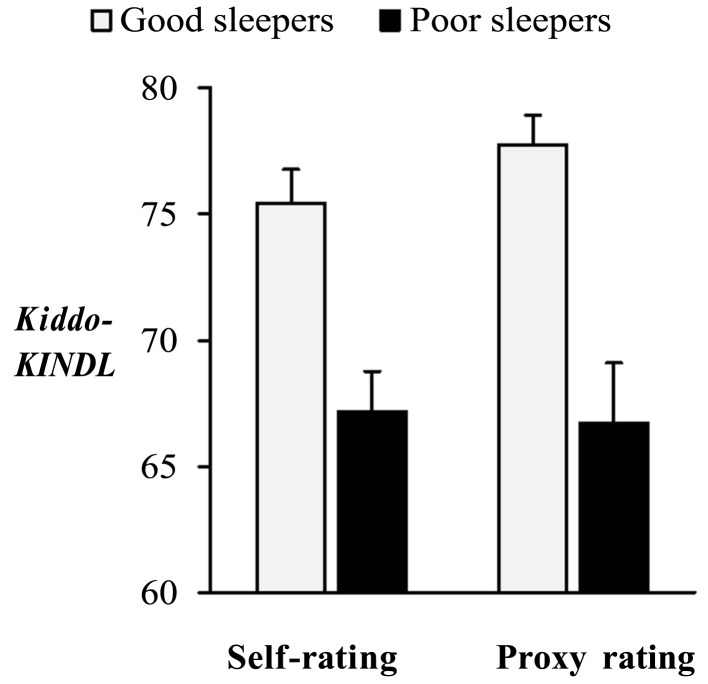
**KINDL-scores in good and poor sleepers**. Good sleepers reported significantly higher *Kiddo-KINDL*-scores than poor sleepers. The same result was found when groups of good and poor sleepers were defined by self- as well as by parental ratings of sleep disturbances. Error bars indicate standard error.

Demographic differences between good and poor sleepers were not existent in the present sample, except for sex (Table 3). Good sleepers as classified by self-rated *SDSC-*values had significantly higher *KINDL-*scores *(M* = 75.43, SD = 8.40) than poor sleepers [M = 67.18, SD = 11.55, *F*_(1, 88)_ = 9.28*, p* < 0.01, Figure 1]. Similarly, good sleepers as classified by parents’ *SDSC*-values had higher *KINDL*-scores (*M* = 77.75, SD = 8.62) than poor sleepers [*M* = 66.71, SD = 11.93, *F*_(1, 88)_ = 21.39, *p* < 0.01, Figure 1]. In stepwise regressions including sex and *SDSC*-score as predictors of *KINDL*-score, sex did not significantly predict *KINDL-*score (self-ratings: β = 0.19, ns.; proxy ratings: β = 0.04, ns.) but *SDSC*-score did (self-ratings: β = −0.30, *p* < 0.01; proxy ratings: β = −0.47, *p* < 0.01).

**Table 3:** **Group differences on demographic variables**.

	Parents’ ratings			Self-ratings		
	Good sleepers	Poor sleepers	Test statistic	Good sleepers	Poor sleepers	Test statistic
Age *M* (SD)	13.61 (±1.37)	13.85 (±1.29)	*F*_(1, 90)_ < 1, ns.	13.69 (± 1.34)	13.66 (±1.36)	*F*_(1, 90)_ < 1, ns.
Sex (female) *n* (%)	30 (45.46)	20 (76.92)	$\chi_{\left(1\right)}^{2}$ = 12.05, *p* < 0.01	13 (33.33)	37 (69.81)	$\chi_{\left(1\right)}^{2}$ = 12.05, *p* < 0.01
Body Mass Index *M* (SD)	20.28 (±3.06)	19.84 (±2.02)	*F*_(1, 78)_ < 1, ns.	19.79 (± 3.21)	20.45 (±2.86)	*F*_(1, 78)_ < 1, ns.
Psychiatric disease *n* (%)	2 (3.03)	1 (3.85)	$\chi_{\left(1\right)}^{2}$ = 0.04, ns.	1 (2.56)	2 (3.77)	$\chi_{\left(1\right)}^{2}$ = 0.10, ns.
Physical disease *n* (%)	4 (6.06)	2 (7.69)	$\chi_{\left(1\right)}^{2}$ = 0.08, ns.	3 (7.69)	3 (5.66)	$\chi_{\left(1\right)}^{2}$ = 0.15, ns.
Medication *n* (%)	5 (7.58)	3 (11.54)	$\chi_{\left(1\right)}^{2}$ = 0.37, ns.	4 (10.26)	4 (7.55)	$\chi_{\left(1\right)}^{2}$ = 0.21, ns.
Single-parent family *n* (%)	18 (27.69)	11 (45.83)	$\chi_{\left(1\right)}^{2}$ = 2.63, ns.	10 (27.03)	19 (36.54)	$\chi_{\left(1\right)}^{2}$ = 0.89, ns.

### Self- and proxy ratings

**Figure 2:**
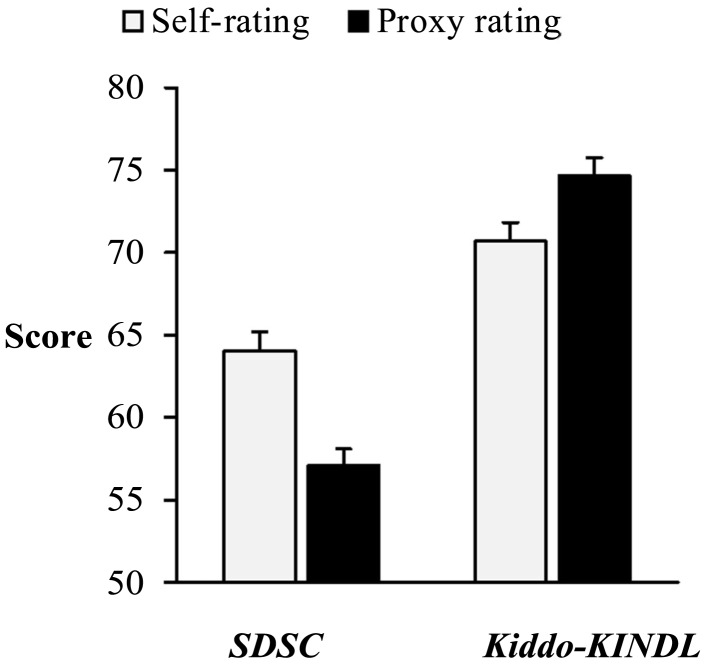
**KINDL and SDSC T-scores in self- and proxy ratings**. Parental ratings of sleep disturbances measured with the *SDSC* were significantly lower than self-rated sleep problems measured with the same instrument. In contrast, *Kiddo-KINDL*-scores rated by proxy were significantly higher than self-reported scores. Both results can be depicted in the same figure, because both scores range from 0 to 100 on an interval scale. Error bars indicate standard error.

*SDSC*-scores were significantly higher in self- (*M* = 64.02, SD = 11.18) than in proxy ratings [*M* = 57.07, SD = 9.74, *t*_(91)_ = 7.00, *p* < 0.01, Figure 2]. Self-rated *KINDL-*scores (*M* = 70.68, SD = 11.07) were significantly lower than those of the proxy rating [*M* = 74.63, SD = 10.83, *t*_(91)_ = 4.97, *p* < 0.01, Figure 2]. For both measures, differences between children’s and parents’ ratings were uncorrelated with age (*SDSC*: *r* = 0.00, ns.; *KINDL*: *r* = −0.13, ns.). The degree of discrepancy in *SDSC*-scores did not differ between boys (*M* = 4.10, SD = 7.66) and girls [*M* = 6.38, SD = 6.77, *t*_(90)_ = 1.52, ns.], but for the *KINDL*-scores, the discrepancy between children’s and parents’ ratings was significantly higher in girls (*M* = −5.54, SD = 8.30) than in boys [*M* = −2.06, SD = 6.33, *t*_(89.22)_ = −2.28, *p* < 0.05].

### Sex differences

Girls scored significantly higher on self- (*M* = 67.28, SD = 11.29), and proxy *SDSC*-ratings (*M* = 59.02, SD = 10.93) than boys [*M* = 60.14, SD = 9.83, *F*_(1, 90)_ = 10.25, *p* < 0.01 for self-ratings; *M* = 54.74, SD = 7.59, *F*_(1, 90)_ = 4.59, *p* < 0.05 for proxy ratings]. Self-rated *KINDL-*scores in girls (*M* = 67.71, SD = 11.60) were lower than in boys [*M* = 74.21, SD = 9.36, *F*_(1, 90)_ = 8.52, *p* < 0.01]. No difference between girls and boys was found in the parental HRQoL assessment [*F*_(1, 90)_ = 1.79, ns.]. Age as a covariate did not significantly influence these results [all *F*_(1, 89)_ ≤ 2.28, ns.].

## Discussion

The aim of the present study was to explore the relationship of sleep quality and HRQoL in adolescents on the basis of self- and proxy ratings. We expected a negative relationship between sleep problems and HRQoL. Moreover, we investigated differences between self- and proxy ratings with respect to the degree of observability for separate subscales.

In line with our first hypothesis, we found a significant positive correlation between sleep quality and HRQoL, indicating that better sleep was associated with higher HRQoL. The group of good sleepers reported significantly higher HRQoL than poor sleepers. Through our statistical analyses, confounding effects of sex, age, or health status on this result were ruled out. Thus, like other diseases and chronic conditions, such as diabetes and asthma (Varni et al., 2007), migraine (Powers et al., 2003), obesity (Schwimmer et al., 2003), or chronic pain (Hunfeld et al., 2001), also sleep problems are accompanied by impaired QoL. Admittedly, the association of sleep disturbances and QoL has already been shown, especially in adults (Iliescu et al., 2003; Yoshimura et al., 2009; Eyigor et al., 2010) and in children (Hiscock et al., 2007; Quach et al., 2009). But in most cases, data for adolescents are unsatisfactory, including large age groups or parental judgments only (Hart et al., 2005; Sung et al., 2008; Ertan et al., 2009). Our study extends the knowledge on the relation between sleep quality and HRQoL in adolescents as it includes self- and proxy ratings and focuses on the group between the ages of 11 and 17. Within this range, all our results were independent of participants’ exact age.

Notably, we found significantly different levels of HRQoL in relation to sleep quality in a non-clinical sample. Therefore, even subclinical sleep problems in adolescence seem to go along with reduced HRQoL. Although HRQoL is influenced by many different characteristics and circumstances, adolescent sleep quality accounted for more than 10% of HRQoL-variance in self-ratings and even more than 20% in proxy ratings. This emphasizes the relevance of untroubled sleep for being healthy and contented. A possible mechanism underlying this association is that sleep is directly related to daytime functioning. Daytime impairments resulting from chronically disturbed sleep include daytime fatigue, mood changes, performance decrements, irritability, memory difficulties, increased environmental sensitivity, and difficulties in coping with everyday life (Moul et al., 2002; Buysse et al., 2005). Thus, social, emotional, physical, and academic aspects of life are affected and the impact of sleep problems can be considered pervasive (Carey et al., 2005).

Furthermore, our results call on parents, educators, and physicians to pay attention to adolescents’ sleep quality. In our sample, over 50% showed indications of sleep disturbances. According to our hypotheses, agreement of self- and parental ratings was especially low for sleep-related behaviors that were not easily observable by proxy (e.g., sleep hyperhidrosis or disorders of arousal and nightmares). These phenomena occur during the night when parents are usually not in immediate proximity to their child. Correlations for more obvious aspects, such as disorders of initiating sleep or excessive somnolence, were at least moderate. We found striking dissimilarity regarding the group size of poor sleepers depending on whose ratings were applied for classification. The number of poor sleepers classified by parental ratings was about 50% smaller than the number of poor sleepers classified by self-reports. If we had relied on proxy reports only, half of the adolescents, who considered their sleep impaired, would have remained unnoticed.

As expected, our results also revealed a significant overestimation of adolescent HRQoL by the parents, especially in girls. This finding replicated previous results, for example those of the BELLA-study (Bullinger et al., 2008). But, in contrast to other studies (Jokovic et al., 2004; Erhart et al., 2009), we found a high correlation between self- and proxy ratings in the *Kiddo-KINDL*. Again, correlations on the subscales varied with respect to the accessibility of the domains. For example, parents and their children shared a more similar view on the adolescent’s physical well-being and family life than on his/her emotional well-being and interactions with friends.

In line with Ravens-Sieberer et al. (2008), girls in our sample showed significantly lower HRQoL compared to boys in the self-rating. Parental ratings, however, revealed no sex differences. Girls also scored higher than boys with respect to sleep problems. The prevalence of sleep disorders may be higher in girls (Johnson et al., 2006), but contradictory findings also exist (Morrison et al., 1992). Alternatively, girls in our sample might have admitted problematic sleep and reduced contentment more easily than boys.

### Limitations

Several limitations of our study have to be considered. Firstly, our sample was recruited in a large city and might not be representative for families from less urbanized regions. Secondly, the recruitment via public institutions offering leisure facilities might have caused selection biases. Education and social integration are likely to be above average in our sample. Probably, more advanced communication patterns between parents and children are also existent, leading to high agreements on the HRQoL-ratings. The third limitation is that data were not acquired in a standardized environment. Biases regarding the presents of distractors or parental influence on adolescents’ answers cannot be ruled out. Fourthly, objective sleep parameters, for example measured with actigraphy, would have been desirable. We do not know whether and to what extent the subjective ratings by parents and adolescents deviate from objective measures. Fifthly, apart from chronological age, further studies should also assess developmental age, because puberty status might affect both sleep and HRQoL. Finally, causal interpretations on the direction of the relationship of sleep and HRQoL cannot be drawn from our results. In our cross-sectional design, sleep problems may either cause or result from poor HRQoL. Although we controlled for the influence of several demographic variables on our data, an unidentified third variable, which accounts for the relationship, may exist.

### Conclusion

Despite the limitations, our results affirm a high prevalence of sleep disturbances in adolescents and, at the same time, the importance of considering both self- and proxy ratings. Furthermore, they highlight the positive relationship between sleep quality and HRQoL. One may speculate that methods to improve sleep in adolescents could also improve HRQoL. This presumption is strongly supported by findings of Schlarb et al. (2011), who have found a significant increase in adolescents’ emotional well-being after a multimodal program for treatment of insomnia. Future studies evaluating sleep-related interventions should therefore include QoL as an outcome variable. Research should also include prospective studies to verify the direction of the association between sleep and HRQoL. Ideally, representative samples should be assessed longitudinally and objective data should be measured in addition to subjective reports.

## Conflict of Interest Statement

The authors declare that the research was conducted in the absence of any commercial or financial relationships that could be construed as a potential conflict of interest.
